# Multiple cardiac malformations in a calf

**DOI:** 10.1111/avj.70141

**Published:** 2026-09-13

**Authors:** LA Soares, IC Matias, DRS Garcia, HS Oliveira Filho, SM Figueiredo, GX Medeiros, LÂ Maia, GJN de Galiza

**Affiliations:** ^1^ Postgraduate Program in Animal Science and Health Federal University of Campina Grande (UFCG) Patos Brazil; ^2^ Veterinary Laboratory João Pessoa Brazil; ^3^ Postgraduate Program in Animal Science Federal University of Paraíba (UFPB) Areia Brazil; ^4^ Department of Veterinary Medicine Federal Institute of Education, Science and Technology of Paraíba (IFPB) Sousa Brazil; ^5^ Department of Veterinary Medicine UFCG Patos Brazil

**Keywords:** calf, cardiovascular malformation, congenital defects, *ectopia cordis*, heart

## Abstract

This paper describes the morphopathological aspects of a case of multiple cardiac malformations in a calf. A two‐day‐old male calf of undefined breed was born with an ectopic heart, presenting with dyspnoea and in lateral recumbency. The owner had repositioned the exposed heart beneath the adjacent skin, which was suclosed, without additional incisions or suturing of the heart or pericardial sac. The animal died the following day and was submitted for necropsy. Macroscopically, the heart was observed to be ventrally pendulous, covered by the pericardial sac, external to the thoracic cavity, with a passage between the manubrium of the sternum and the middle scalene muscle (*ectopia cordis*). The heart was rounded, and three cardiac chambers were observed: the right ventricle, left ventricle and a single dilated atrial cavity located above the left ventricle, with absence of the atrial septum and a rudimentary right ventricle. The atrial cavity was dilated with rudimentary pectinate muscles. A caudal vena cava and pulmonary veins emptied into the single atrial cavity. In the right chamber of the heart, there was marked myocardial thickening with a rudimentary ventricular space, a poorly developed tricuspid valve, absence of a pulmonary trunk, a single‐inlet univentricular atrioventricular connection and a single‐outlet ventriculoarterial connection. The occurrence of *ectopia cordis* associated with cardiac malformations is a rare condition incompatible with the life of calves.

Congenital heart malformations are uncommon in animals and humans.^1^, ^2^ Among the congential cardiac malformations that occur in ruminants, ventricular septal defect, *ectopia cordis*, right ventricular hypoplasia, aortic dextraposition, valvular hematomas, patent foramen ovale and endocardial fibroelastosis have been reported.^1^, ^3^

Defects in atrial and ventricular septa are the most prevalent malformations in cattle,^4^, ^5^ while *ectopia cordis* is a rare and poorly described condition.^6^ *Ectopia cordis* is characterised by the development of the heart outside the thoracic cavity.^7^ It is classified according to the location of the organ in the cervical, cervicothoracic, thoracic, thoracoabdominal and abdominal regions.^8^, ^9^ In cattle, most described cases of *ectopia cordis* are of the cervical type.^10^, ^11^

Cases of *ectopia cordis* are associated with other cardiac defects in cattle, such as double apex,^8^, ^9^, ^10^, ^12^ tricuspid valve dysplasia,^13^ patent ductus arteriosus,^8^ aortic stenosis and bilateral concentric cardiac hypertrophy.^14^ However, there are no studies describing cervical *ectopia cordis* associated with multiple atrial, ventricular and vascular defects in cattle. Therefore, the aim was to describe the morphological and pathological aspects of a case of multiple cardiac malformations in a calf.

## Case report

A two‐day‐old male calf of no defined breed was born with its heart outside the thoracic cavity, not covered by skin, and presenting with dyspnoea and inability to rise. The animal belonged to a herd of approximately 40 cattle. The herd was raised in a semi‐intensive system, consuming corn bran and native pasture with black jurema (*Mimosa tenuiflora*) and pereiro (*Aspidosperma pyrifolium*); however, the consumption of these plants by the animals was not reported. The calf was the product of the crossing of a bull recently introduced into the herd, and there was no history of malformations in the herd. Both parents were of undefined breed. The dam was approximately 4 years old, and this was her second parturition. The calf was a singleton, and breeding was by natural mating. According to the owner, the externally exposed heart had been repositioned beneath the adjacent skin, which was sutured closed, without additional incisions or suturing of the heart or pericardial sac. The procedure was performed without veterinary assistance. The animal died the following day and was submitted for necropsy.

Macroscopically, an increase in fluid volume was observed in the ventrocranial region of the thoracic cavity, with the presence of a skin suture measuring approximately nine centimeters in length. Upon retracting the skin, the heart was positioned ventrally and pendulous, outside the thoracic cavity, located between the manubrium of the sternum and the middle scalene muscle. The heart was covered by the pericardial sac and rounded with a poorly defined apex and interventricular sulci (paraconal and subsinuous). The left atrium was poorly developed and the right atrium rudimentary (Figure 1(A)). Upon opening the heart, three cardiac chambers were observed, including the right ventricle (2.0 × 1.8 × 0.7 cm), left ventricle (3.5 × 5.0 × 2.5 cm) and a single atrial cavity (9.0 × 8.3 × 5.4 cm) located above the left ventricle. This cavity was dilated with rudimentary pectinate muscles and absence of an atrial septum (Figure 2(A)). The caudal vena cava and pulmonary veins emptied into the single atrial cavity (Figure 1(B)). The bicuspid valve was slightly thickened and separated the single atrial cavity from the left ventricle. The aortic semilunar valve and the aorta were normal. Adjacent to the semilunar valve, a communicating orifice (0.8 cm in diameter) was observed in the left ventricle, from which an artery emerged with anastomosis to the brachiocephalic trunk (Figures 2(B) and 3(A)).

**Figure 1:**
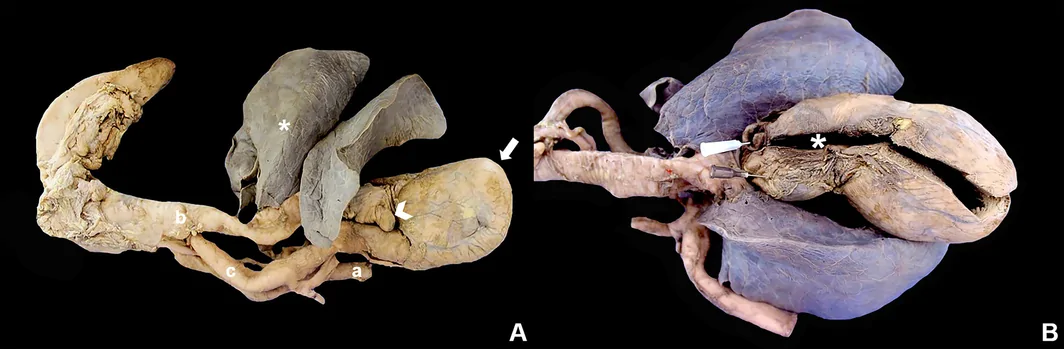
Cardiac malformations in a calf. (A) Cardiorespiratory tract with a rounded heart with an apex (arrow) and poorly defined paraconal and subsinuous interventricular sulci and a poorly developed left atrial projection (arrowhead). Lungs with slight bilateral hypoplasia (*). Esophagus (A), trachea (B) and aorta and its branches (C) unaltered. B. Right heart with caudal vena cava (gray needle) and pulmonary veins (white needle), both emptying into a dilated and single atrial cavity (*).

**Figure 2:**
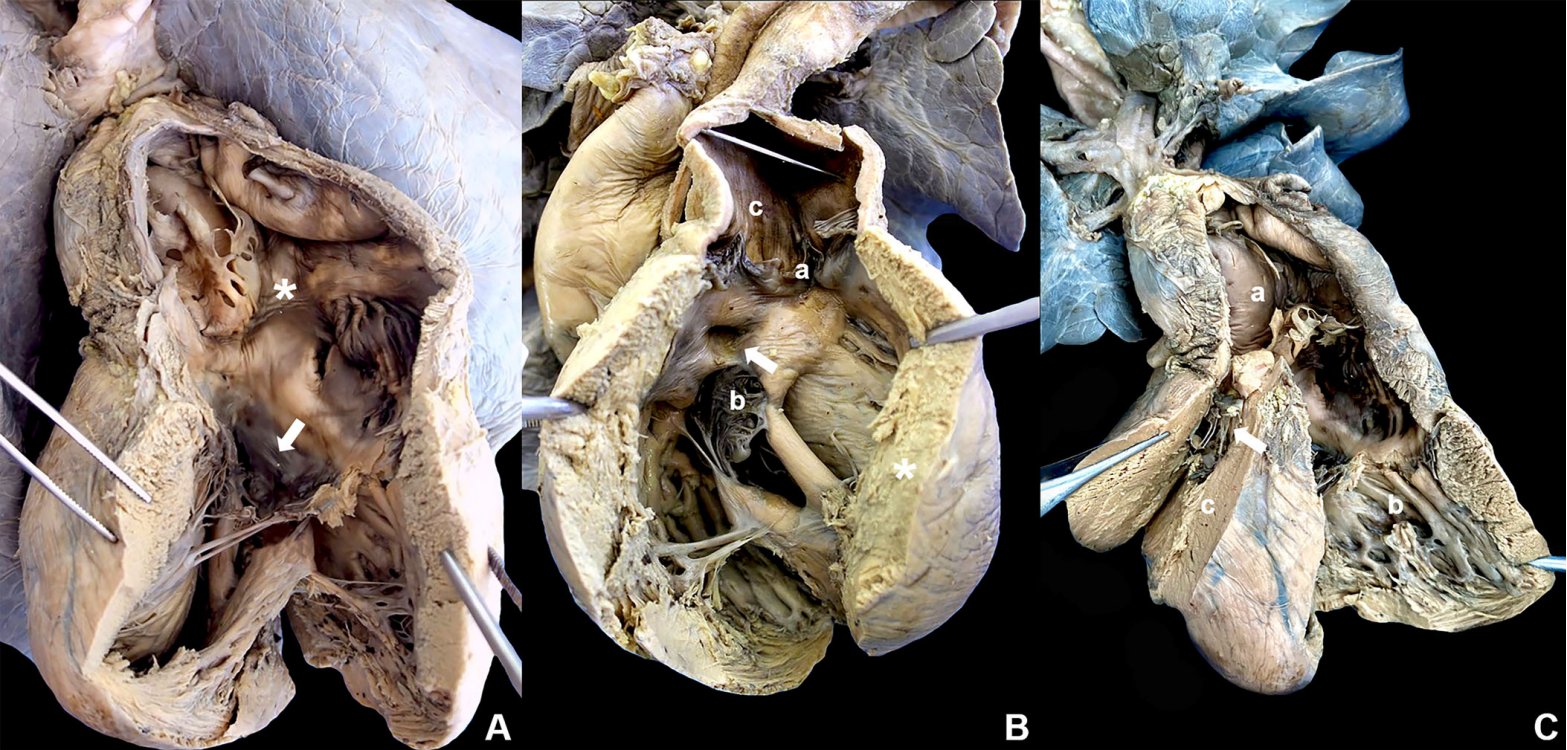
Cardiac malformations in a calf. (A) Left heart with a single, dilated atrial cavity with rudimentary pectinate muscles and absence of an atrial septum (*). Left atrioventricular valve (arrow) separating the single atrial cavity from the left ventricle. B. Left heart with a slightly thickened ventricular wall (*) and a communicating orifice through which an artery emerges at the base of the heart (arrow), adjacent to the semilunar aortic valves (A), the left atrioventricular valve (B) and the aorta (C). C. Heart with a single atrial cavity (A), left ventricle (B) and right ventricle with marked myocardial thickening with a rudimentary ventricular space (C) and a poorly developed tricuspid valve (arrow).

**Figure 3:**
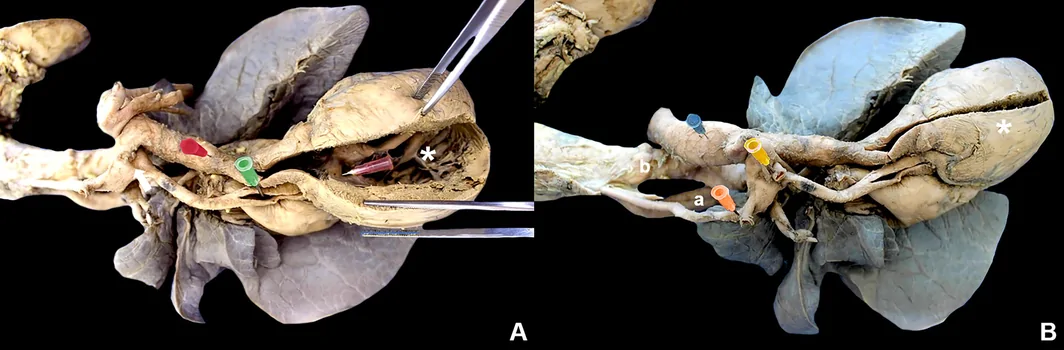
Cardiac malformations in a calf. (A) Left heart (*) with an artery (green needle) adjacent to the aorta (red needle) that empties into an opening in the left ventricle (pink needle) and anastomoses with the brachiocephalic trunk (arrow). B. Left heart (*) with large vessels of the brachiocephalic trunk (yellow needle), aorta (blue needle) and coronary artery (orange needle), adjacent to the esophagus (A) and trachea (B).

In the right heart, marked thickening of the myocardium was observed (wall thickness: 2.5 cm) with a rudimentary ventricular space (2.0 × 1.8 × 0.7 cm), a poorly developed tricuspid valve (remnant length: 1.6 cm), absence of the pulmonary trunk, single inlet atrioventricular connection and single outlet ventriculoarterial connection. The left ventricle (3.5 × 5.0 × 2.5 cm) had a wall thickness of 1.7 cm. The mitral valve measured 3.7 cm in length and 0.2 cm in thickness, and the aorta measured 2.4 cm in diameter. The right and left coronary arteries remained normal (Figure 3(B)). Lung irrigation was performed via branches of the aortic arch. Figure 4 shows a schematic drawing of the main cardiac defects observed in the cardiac chambers and blood vessels of this case. The sternum presented manubrium, sternebrae and xiphoid processes without macroscopic alterations. Additionally, slight bilateral pulmonary hypoplasia was observed. No other significant macroscopic or microscopic lesions were observed in the abdominal organs or central nervous system.

**Figure 4:**
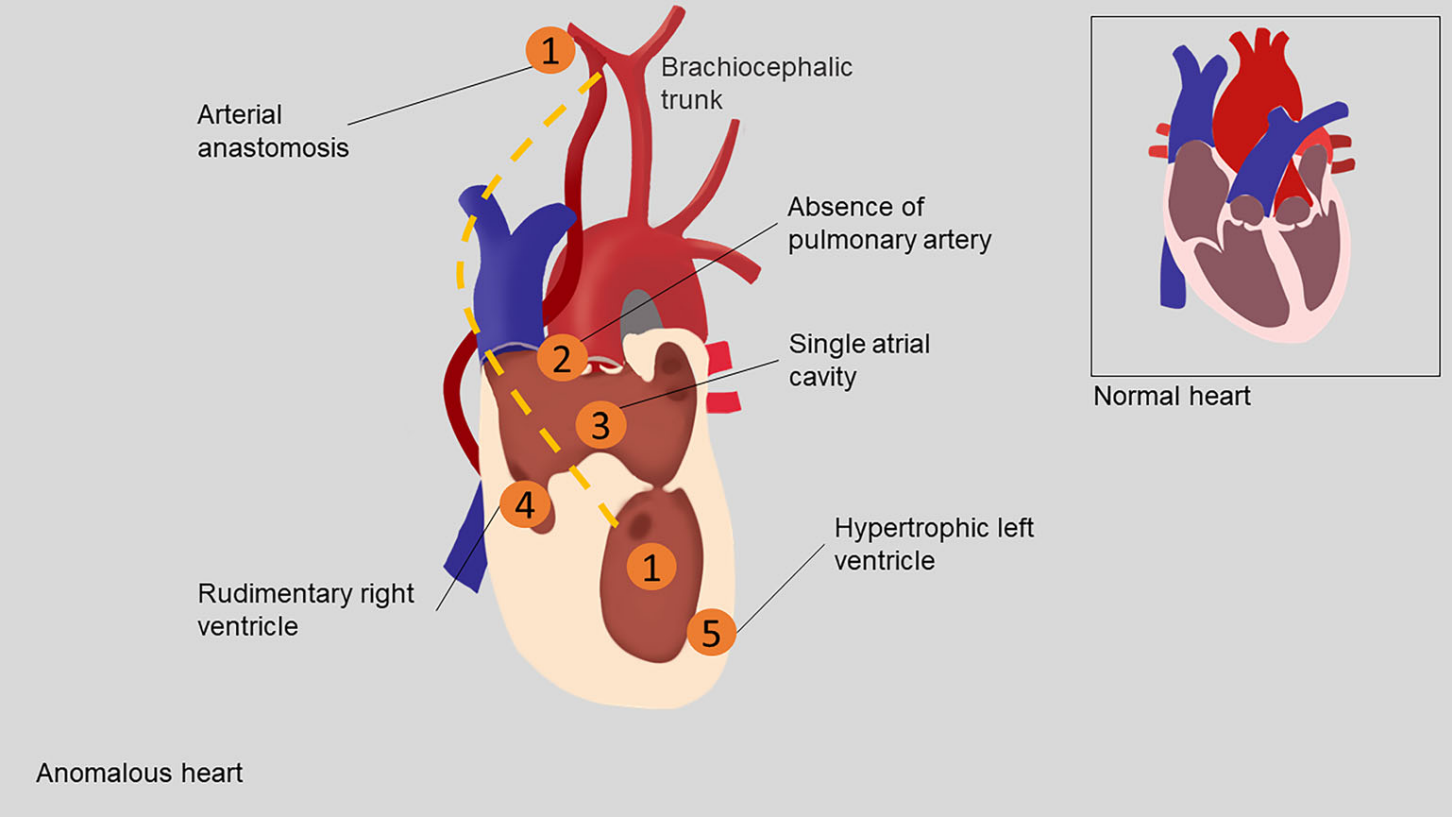
Schematic drawing showing the main congenital defects observed in the heart chambers and blood vessels of the calf. 1. Arterial anastomosis with communication to the left ventricle. 2. Absent pulmonary trunk. 3. Single cavity with interatrial communication. 4. Rudimentary right ventricle. 5. Slight hypertrophy of the left ventricular wall. Inset: Normal representation of the heart.

## Discussion

Based on the anatomopathological findings, cervical *ectopia cordis*, atrial septal defect, pulmonary trunk agenesis, tricuspid aplasia and right ventricular hypoplasia were identified. During heart development, there are three arteriovenous communications: between atria (through the atrial septum and foramen ovale), between ventricles (ventricular septum) and between great vessels (ductus arteriosus), with closure of the septa during intrauterine life.^15^ When cardiac communications do not close, blood can be improperly conducted between the right and left heart chambers, developing congenital heart defects.^7^ In this case, it was not possible to determine whether the animal had heart failure before death; however, it should be noted that the identified cardiovascular anomalies were severe and resulted in significant functional impairment of circulatory function after birth. In live calves, cardiovascular abnormalities may be associated with clinical signs such as dyspnoea, weakness, exercise intolerance, cyanosis and recumbency.^1^

During fetal development, the separation of the atria begins with the growth of the *septum primum*, which grows towards the atrioventricular junction, which together with the endocardial cushions forms the atrioventricular valves and separates the ventricles.^16^ In the failure of fusion of the *septum primum* with the endocardial cushions, there is no separation of the atria and there is no formation of the *septum secundum* that will give rise to the foramen ovale,^17^ causing an atrial septal defect and the formation of a single complete atrial cavity, as observed in this case.

Arteriovenous communications are frequently associated with conotruncal septal anomalies during uterine development, resulting in cardiovascular defects such as pulmonary artery stenosis, interventricular septal defect, dextroposition of the aorta and right ventricular hypertrophy, which when associated characterise Tetralogy of Fallot.^18^, ^19^ In this case, the calf did not have this anomaly, since no interventricular septal defect was observed. However, it is noteworthy that the absence of the pulmonary trunk is a rare congenital defect and is commonly associated with other cardiac malformations,^20^ but without descriptions in calves.

*Ectopia cordis* can occur in isolation or associated with congenital heart defects and in other organs.^14^, ^21^ In calves, dilated atrial chamber, double apex, double vena cava, double azygos vein and sternal anomalies are the anatomical defects frequently reported and associated with *ectopia cordis*.^12^ However, it is noteworthy that there are no descriptions of the absence of the foramen ovale with single atrial formation, agenesis of the pulmonary trunk and tricuspid aplasia associated with ectopic heart in cattle.

Animals with *ectopia cordis* can survive for a short time to years, with cervical cases generally being compatible with life.^8^, ^13^ In contrast, calves with other types of *ectopia cordis* die before or shortly after birth,^22^ especially when associated with cardiac or extra‐cardiac defects that are not compatible with life.^8^, ^12^, ^14^, ^21^

The pathogenesis of ectopic heart is poorly understood, but it is believed to occur due to failure of midline fusion of the mesoderm and alteration of the embryonic disc^23^; midline failure resulting in rupture of the chorionic sac and amniotic band syndrome.^24^ In cervical *ectopia cordis*, the heart may or may not be covered by the pericardial sac and skin.^10^, ^25^ In this case, only pericardial sac covering was observed, similar findings described by other authors.^12^, ^26^ It is believed that a heart devoid of skin covering becomes more vulnerable to trauma and infection, contributing to decreased animal survival.^6^

The causes of congenital heart defects in ruminants are unknown.^1^, ^25^ However, intrinsic and extrinsic factors such as genetic or chromosomal abnormalities, contact with chemicals, ingestion of teratogenic plants,^27^, ^28^ radiation and infectious agents have been identified as causes of congenital defects.^1^, ^14^

Structural chromosomal alterations are generally associated with multiple malformations, resulting from genetic fragmentation and possibly related to extrinsic environmental factors.^19^ Most hereditary congenital defects are transmitted by sporadic autosomal recessive genes, without a related cause, resulting in the birth of defective animals and normal progenitors.^29^ Since no relationship with other causes was observed in this case, it is assumed that the malformations may be related to occasional chromosomal alterations of autosomal recessive genes; however, confirmation would require crossbreeding tests or genetic sequencing.

The occurrence of *ectopia cordis* associated with cardiovascular malformations is a rare and life‐threatening condition. The cardiac defects and the extent of the lesions directly interfered with the survival of the affected animal. In this case, it was not possible to determine the factors related to the development of congenital defects, and further investigations are needed to determine the aetiologies of cardiac malformations in cattle, such as genetic and genomic sequencing.

## Statement of consent

The owner provided informed verbal consent for the animal's clinical, pathological and related data to be used for scientific purposes and publication in this case report.

## Conflicts of interest or sources of funding

The authors declare no conflict of interest.

## Data Availability

The data supporting the results of this study are available from the corresponding author upon reasonable request.

## References

[1] Constable PD , Hinchcliff KW , Done SH et al. Diseases of the cardiovascular system. In: Constable PD , Hinchcliff KW , Done SH , Grunberg W , editors. Veterinary medicine: a textbook of the diseases of cattle, horses, sheep, pigs and goats. 11th edn. Saunders, St. Louis, 2016;704.

[2] Sadlecki P , Krekora M , Krasomski G et al. Prenatally evolving *ectopia cordis* with successful surgical treatment. Fetal Diagn Ther 2011;30:70–72. 10.1159/000326300. 21508620

[3] Gopal T , Leipold HW , Dennis SM . Congenital cardiac defects in calves. Am J Vet Res 1986;47:1120–1121. 10.1002/vetr.701. 3717736

[4] McManus A , Moloney T , Kelly P et al. An unusual presentation of developmental anomalies of the cardiovascular system including tetralogy of Fallot, double outlet right ventricle, patent foramen ovale and persistent right aortic arch in a Friesian calf. BMC Vet Res 2020;16:224. 10.1186/s12917-020-02439-8. PMC7325375 32605568

[5] Wronski JG , Cecco BS , Lorenzett MP et al. Major cardiac defects in cattle in southern Brazil: retrospective study of necropsies over 20 years (2000–2019). Cienc Rural 2021;51:e20201106. 10.1590/0103-8478cr20201106.

[6] Cerqueira LA , Macedo IL , Sousa DER et al. Complete thoracic *ectopia cordis* in two lambs. Animals (Basel) 2024;14:1–8. 10.3390/ani14152213. PMC11310945 39123739

[7] Ocarino NM . Sistema cardiovascular. In: Santos RL , Alessi AC , editors. Patologia veterinária. 3rd edn. Roca, Rio de Janeiro, 2022.

[8] Hiraga T , Abe M . Eight calves of cervical *ectopia cordis* and their sternums. Jpn J Vet Sci 1986;48:1199–1206. 10.1292/jvms1939.48.1199. 3820919

[9] Shirian S , Oryan A , Samadian MR . *Ectopia cordis* in a male Holstein Friesian calf. Open Anat J 2010;2:34–36. 10.2174/1877609401002010034.

[10] Hamali H , Ashrafihelan J . A report on bovine fetal *ectopia cordis* cervicalis associated with two cervical sacs. Iran J Vet Res 2010;11:287–290. 10.22099/IJVR.2010.146.

[11] Ozer K , Aydin D , Guzel O et al. *Ectopia cordis* cervicalis and its surgical treatment in a Holstein calf. Kafkas Univ Vet Fak Derg 2014;20:469–472. 10.9775/kvfd.2013.10254.

[12] Hiraga T , Abe M , Iwasa K et al. Cervico‐pectoral *ectopia cordis* in two Holstein calves. Vet Pathol 1993;30:529–534. 10.1177/03009858930300060. 8116146

[13] Onda K , Sugiyama M , Niho K et al. Long‐term survival of a cow with cervical *ectopia cordis* . Can Vet J 2011;52:667–669. PMC3095170 22131586

[14] Pusta DL , Sobolu R , Bodea A et al. Cervical cardiac ectopia in cattle: a case report. Vet Med (Praha) 2015;72:314–319. 10.15835/buasvmcn-vm:11444.

[15] Hyttel P . Desenvolvimento das células do sangue, do coração e do sistema vascular. In: Hyttel P , Sinowatz F , Vejlsted M , editors. Embriologia veterinária. 1st edn. Elsevier, Rio de Janeiro, 2012;182.

[16] Moore KL , Persaud TVN , Torchia MG . Sistema cardiovascular. In: Moore KL , Persaud TVN , Torchia MG , editors. Embriologia clínica. 10th edn. Elsevier, Rio de Janeiro, 2022;294–302.

[17] Robinson WF , Robinson NA . Cardiovascular system. In: Maxie MG , editor. Jubb, Kennedy, and Palmer's pathology of domestic animals. 6th edn. Vol. 3. Elsevier, ST. Louis, MO, 2016.

[18] Mendes LCN , Borges AS , Luvizotto MCR et al. Tetralogia de Fallot em bovinos. Arq Bras Med Vet Zootec 1999;51:35–38.

[19] Sadler TW . Desenvolvimento do coração. In: Langman embriologia médica. 14th edn. Guanabara Koogan, Rio de Janeiro, 2021;586.

[20] Malani SK , Kashyap P , Nalawade D . A rare case of unilateral agenesis of left pulmonary artery presenting as severe pulmonary arterial hypertension. Glob Cardiol Sci Pract 2023;3:15. 10.21542/gcsp.2023.15. PMC10422877 37575286

[21] Sanchez GA , Martinez MM , Oviedo ST et al. *Pectus excavatum* associado a *ectopia cordis* en un neonato bovino. Rev MVZ Cordoba 2005;10:684–688. 10.21897/rmvz.472.

[22] Eroksuz H , Metin N , Eroksuz Y . Total pectoral ectopia cordis and other congenital malformations in a calf. Vet Rec 1998;142:437. 10.1136/vr.142.16.437-a. 9595636

[23] Sutaria TV , Sutaria P , Panchasara HH . *Ectopia cordis* in a Mehsana kid: a case report. Indian J Small Rumin 2014;20:136–137.

[24] Abi B , Giamberti A . *Ectopia cordis*: a case report and review of related embryology. In: Winter scientific meeting of the British Association of Clinical Anatomy. University of East Anglia; 2008, Norwich, 2008. [in press].

[25] Ramadan RO , Abdin‐Bey MR . *Ectopia cordis* in a goat. Reprod Domest Anim 1993;28:137–139. 10.1111/j.1439-0531.1993.tb01164.x.

[26] Frackowiak H , Jaskowski JM , Nabzdyk M et al. Congenital sternal *ectopia cordis* in a Limousin calf: a case report. Acta Vet Brno 2014;83:51–54. 10.2754/avb201483010051.

[27] Dantas AFM , Riet‐Correa F , Medeiros RMT et al. Malformações congênitas em ruminantes no semiárido do Nordeste brasileiro. Pesq Vet Bras 2010;30:807–815. 10.1590/S0100-736X2010001000002.

[28] Stegelmeier BL , Davis TZ . In: Rudmann DG , Mahler BW , editors. Poisonous plants. In: Haschek and Rousseaux's handbook of toxicologic pathology. Heinz‐Taheny KM. 4th edn. Vol. 3., Academic Press, San Diego, CA, 2023;489–546.

[29] Schild AL . Defeitos congênitos. In: Riet‐Correa F , Schild AL , Lemos R , Borges JR , Mendonça FS , Machado M , editors. Doenças de ruminantes e equídeos. 4th edn. Vol. 1. Editora MedVet, São Paulo, 2023;3–7.

